# Mapping the therapeutic landscape in emergency incisional hernia: a scoping review

**DOI:** 10.1007/s10029-025-03278-y

**Published:** 2025-02-18

**Authors:** Andrea Carolina Quiroga-Centeno, Sebastian Schaaf, Ana Pilar Morante-Perea, Stavros A. Antoniou, Heather Bougard, Umberto Bracale, Sara Capoccia Giovannini, Eva Deerenberg, René H. Fortelny, Christine Gaarder, Miguel Ángel García-Ureña, Katie Gilmore, Sergio Alejandro Gomez-Ochoa, Ferdinand Köckerling, Maciej Pawlak, Francesca Pecchini, José A. Pereira-Rodriguez, Yohann Renard, Benoît Romain, Elena Schembari, Alexis Theodorou, Cesare Stabilini

**Affiliations:** 1 Department of Surgery, Universidad Industrial de Santander, Bucaramanga, Colombia; 2 School of Translational Medicine, Medical Faculty Mannheim, Heidelberg University, Mannheim, Germany; 3 Department of General, Visceral and Thoracic Surgery, German Armed Forces Central Hospital Koblenz, Rübenacher Str. 170, 56072 Koblenz, Germany; 4 Hospital Universitario Fundación Alcorcón, Madrid, Spain; 5 Medical School, European University Cyprus, Nicosia, Cyprus; 6 Department of Surgery, New Somerset Hospital, University of Cape Town, Cape Town, South Africa; 7 Department of Gastroenterology, Endocrinology and Endoscopic Surgery, University Hospital of Naples Federico II, Naples, 80131 Italy; 8 Department of Surgery, Policlinico San Martino IRCCS, Department of Surgical Sciences, University of Genoa, Genoa, Italy; 9 Department of Surgery, Franciscus Gasthuis en Vlietland, Rotterdam, the Netherlands; 10 Medical faculty, Sigmund Freud Private University Vienna, Vienna, Austria; 11 Institute of Clinical Medicine, Department of Traumatology, University of Oslo, Oslo University Hospital Ulleval, Oslo, Norway; 12 Grupo de Investigación de Pared Abdominal Compleja, Facultad de Medicina, Universidad Francisco de Vitoria. Hospital Universitario del Henares, Carretera Pozuelo-Majadahonda km. 1,800, Pozuelo de Alarcón (Madrid), 28223 Spain; 13 Department of General & Abdominal Wall Surgery, Golden Jubilee National University Hospital, Glasgow, UK; 14 School of Medicine, Dentistry and Nursing, University of Glasgow, Glasgow, UK; 15 Heart Failure and Transplant Clinic, Fundación Cardiovascular de Colombia, Floridablanca, Colombia; 16 Department of General Internal Medicine and Psychosomatics, Heidelberg University Hospital, Heidelberg, Germany; 17 Hernia Center, Vivantes Humboldt-Hospital, Academic Teaching Hospital of Charité University Medicine, Am Nordgraben 2, 13509 Berlin, Germany; 18 Department of General Surgery, Emergency and New Technologies, Baggiovara General Hospital, AOU Modena, Modena, Italy; 19 Abdominal Wall Surgery Unit, Section of General Surgery, Department of General Surgery, Parc de Salut Mar, Hospital del Mar Medical Research Institute (IMIM), Passeig Maritim 25-29, Barcelona, 08003 Spain; 20 Department of General, Digestive and Endocrine Surgery, Reims Champagne-Ardennes, Robert Debré University Hospital, Reims, France; 21 Department of Digestive Surgery, Centre Hospitalier Universitaire de Strasbourg, Strasbourg, France; 22 Department of Colorectal Surgery, Royal Devon and Exeter Hospital, Exeter, UK; 23 Department of Surgery, Hippocratio Hospital, University of Athens, Athens, Greece

**Keywords:** Incisional hernia, Emergency repair, Hernia repair, Surgical mesh, Postoperative complications, Scoping review

## Abstract

**Purpose:**

Incisional hernias (IH) represent common complications following abdominal surgeries, with emergency repair associated with increased morbidity and mortality. This scoping review aimed to map the existing literature on emergency incisional hernia repair, identify research gaps, and inform future guideline development.

**Methods:**

A comprehensive literature search was conducted in PubMed MEDLINE and SCOPUS for studies published between January 2000 and August 2024. Articles addressing any aspect of emergency incisional hernia repair in adults were included. Data extraction focused on study characteristics, patient demographics, surgical approaches, and outcomes.

**Results:**

Of 801 unique articles identified, 73 met the inclusion criteria. Most were cohort studies (73.97%), with only one randomized trial. The primary areas of interest were repair methods (47.95%), operative outcomes (31.51%), risk assessment (16.44%), and diagnosis (5.48%). Pooled analysis revealed a predominantly female (63%), elderly (mean age 62.3 years), and comorbid patient population. The most frequent study endpoints were readmission (18%), surgical site infection (12%), reoperation (8%), and mortality (4%). Significant heterogeneity was observed in defect characterization and surgical techniques.

**Conclusion:**

This review highlights a paucity of randomized studies guiding emergency incisional hernia management. Key issues identified include inconsistent definitions of emergency presentation, limited data on hernia characteristics, and a lack of standardized outcome reporting. Future research should focus on developing a unified classification system for emergency incisional hernias, evaluating the role of imaging in decision-making, and conducting comparative studies on various treatment strategies across different clinical scenarios.

**Supplementary Information:**

The online version contains supplementary material available at 10.1007/s10029-025-03278-y.

## Introduction

Abdominal wall defects represent a major global health burden with increasing incidence [1]. Moreover, incisional hernias (IH), which can occur after every type of surgery accessing the abdominal wall, are common long-term complications that further exacerbate the issue. Despite recent efforts to optimize abdominal wall closure strategies and prevent the development of IH with the use of prophylactic meshes, the incidence varies depending on the surgical approach [2–4]. Average rates of 5–20% are reported after laparotomies, and these rates can be exceeded in cases of open repair of abdominal aortic aneurysms [5–8], obesity, and colorectal surgery [9]. In addition, recurrent incisional hernias account for a relatively large proportion of all incisional hernias, with a rate of around 20–25% [10, 11].

While the elective treatment of IH is a studied field, providing sufficient evidence to give recommendations in guidelines, evidence-based advice for managing the emergency presentation of incisional hernias is still controversial [12]. The urgent surgical management of IH presents formidable challenges, leading to worse outcomes than elective repair [13]. Severe pain and symptoms of acute bowel obstruction typically cause patients to present suddenly in the emergency department. These patients are often frail and have comorbid conditions that cannot be prehabilitated, negatively affecting postoperative outcomes. Additionally, ongoing therapies, such as oral anticoagulation or steroids, which cannot be rapidly antagonized, introduce a higher risk of complications [14, 15].

The major issue in emergency situations is that a localized abdominal wall condition can become a systemic problem, endangering patients’ lives through obstruction and/or strangulation of bowel loops, potentially causing systemic inflammatory response affecting multiple organs. If acute incarceration is diagnosed and manual reduction (taxis) fails or is deemed inappropriate, immediate surgical repair is indicated. Therefore, the timing of surgical intervention is crucial, as progression to systemic inflammatory response and sepsis is challenging to reverse and demands increasing expertise and resources for its effective management [12, 16].

Patients’ stability, bowel obstruction and massive tissue contamination caused by bowel perforation are major determinants in treatment choice, probably representing the most significant challenge for general and abdominal wall surgeons. Depending on the hernia morphology, localization, and patient condition, alternatives to open repair may be considered, subject to local availability and expertise. Additionally, although the use of mesh is the gold standard for preventing recurrences in incisional hernia repair (IHR), its use in contaminated or dirty situations poses a risk of subsequent colonization.

Members of the European Hernia Society (EHS) science wing conducted a comprehensive scoping review to uncover the available body of evidence and provide crucial insights for guideline developers. Focusing on emergency incisional hernia repair, this review aims to map the existing literature, identify research gaps, and inform the development of future guidelines.

## Materials and methods

This scoping review was conducted using the methodological framework proposed by Arksey and O’Malley and further refined by Levac et al. [17, 18].

### Information sources and search strategy

A comprehensive literature search was performed in PubMed MEDLINE and SCOPUS. The search strategy was designed using Medical Subject Heading (MeSH) terms and relevant keywords, to capture all relevant studies and publications on emergency IHR (**Supplementary material**). The search was restricted to articles published in English and included studies from 2000 to August 2024. Additionally, the reference lists of the identified articles were hand-searched to include any additional relevant studies. When full texts could not be obtained through standard methods, the authors were emailed directly for access. Additionally, Clinicaltrials.gov was queried using the keyword “incisional hernia” to identify ongoing clinical trials assessing IH in the emergency setting.

### Eligibility criteria

Inclusion criteria considered studies of any type (original research articles, reviews, case reports, editorials, and letters to the editor) addressing any aspect of emergency IHR in adult patients. Articles were excluded if emergency hernia patients were not characterized. Conference abstracts were reviewed for corresponding full-text publications but were not included in the final analysis. Additionally, studies focusing on parastomal hernias were excluded.

### Data screening and extraction

The initial search results were imported into EndNote 21™ for reference management, and duplicates were removed [19]. Titles and abstracts were screened by three researchers (ACQ, SS, AM) using ASReview (Version 1.6.3) [20], an active learning tool for prioritizing relevant studies. Three authors reviewed full-text articles (ACQ, SS, AM). Disagreements between reviewers were resolved by consensus or with a fourth reviewer (CS). Data extraction was performed using a standardized form in Microsoft Excel, capturing study characteristics (such as first author, year of publication, country, and study design), specificity to emergency incisional hernia repair (versus mixed hernia types or combined emergency and elective surgeries), and the primary focus of the study (risk assessment, diagnosis, repair methods [type of approach, type of repair, mesh position, fascial closure, mesh characteristics], and operative outcomes). Attention specifically on specific populations (cirrhotic, elderly, obese, Chronic Obstructive Pulmonary Disease [COPD], diabetic, smokers, cancer patients, immunocompromised, bleeding disorders, previous hernia repair, multiple hernia defects) was also documented. Outcomes recorded included morbidity, Clavien-Dindo classification, surgical site infection (SSI), surgical site occurrence (SSO), reintervention, readmission, mesh infection, ICU admission, bowel resection, length of stay, quality of life, mortality, and recurrence rates. Additional data included follow-up duration, hernia location (EHS classification), mean hernia size, and evidence level based on the medical evidence pyramid of each study [21].

### Data analysis and reporting

Data analysis and reporting followed the PRISMA-ScR (Preferred Reporting Items for Systematic Reviews and Meta-Analyses Extension for Scoping Reviews) guidelines [22]. A narrative synthesis approach was adopted to integrate and summarize the findings from the included studies, highlighting significant trends, gaps, and inconsistencies. Regarding quantitative analyses, categorical variables were described as frequencies and percentages. Assessment of the risk of bias was not performed, as this aligns with the standard methodology for scoping reviews. A proportions meta-analysis was conducted to quantitatively summarize the prevalence of key outcomes across studies using random-effects models and the Freeman-Tukey double arcsine transformation. Data analysis was performed using R (version 4.4.1). The ‘meta’ package (v4.17-0) was employed for the proportions meta-analysis [23]. Figures were created using Flourish Studio.

## Results

### Study selection

**Fig. 1:**
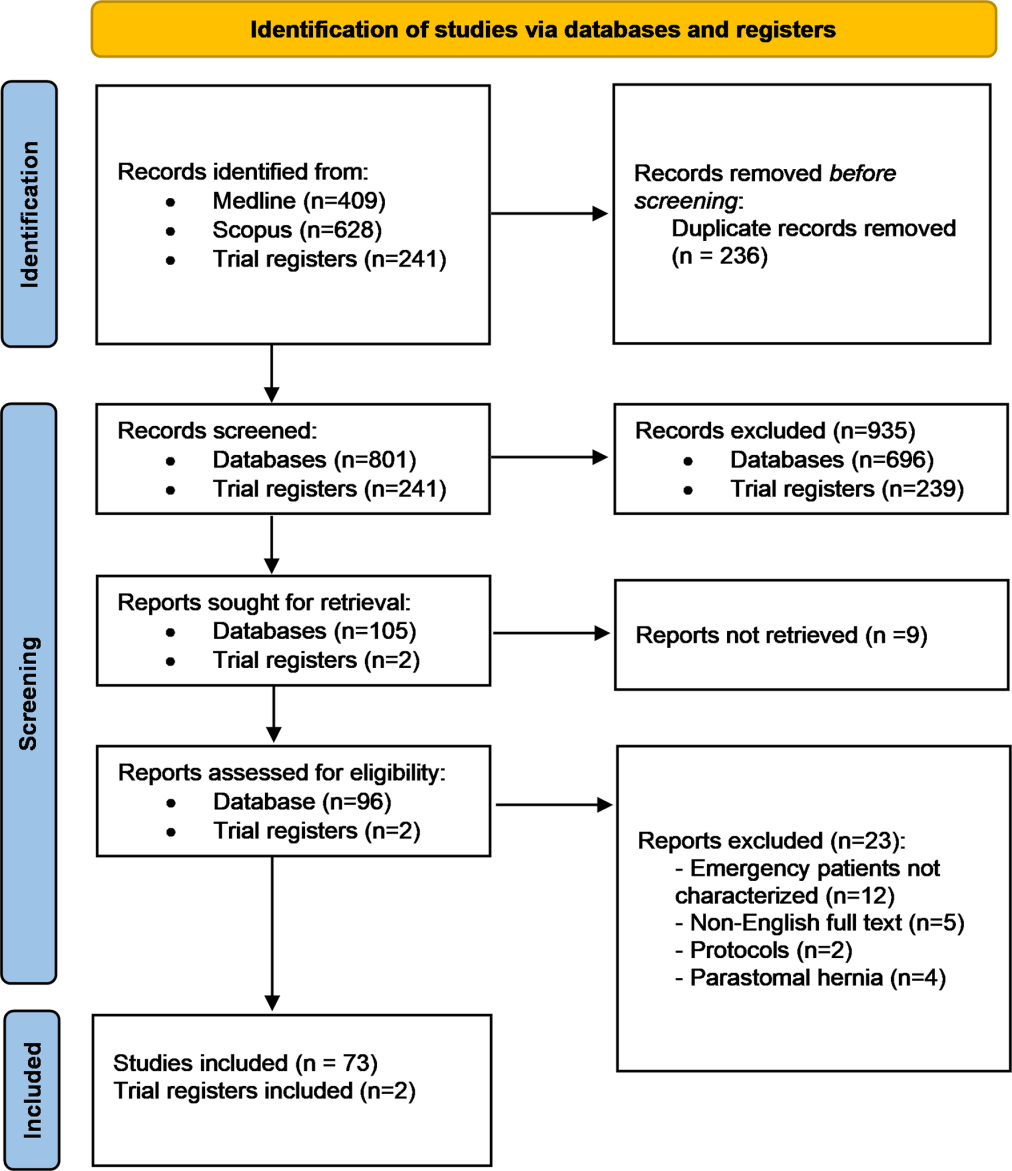
PRISMA flow diagram of the study selection process

The initial search identified 409 articles from Medline and 628 from Scopus. After removing duplicates, 801 unique articles were available for review. Title and abstract screening resulted in 105 articles being selected for full-text review, of which 73 met the inclusion criteria and were incorporated into this scoping review [6, 13, 24–94]. Additionally, 241 entries were retrieved from trial registries, with two ongoing trials addressing incisional hernia repair (IHR) in the emergency setting. The PRISMA flow diagram outlines the study selection process (Fig. 1).

### Study characteristics

**Fig. 2:**
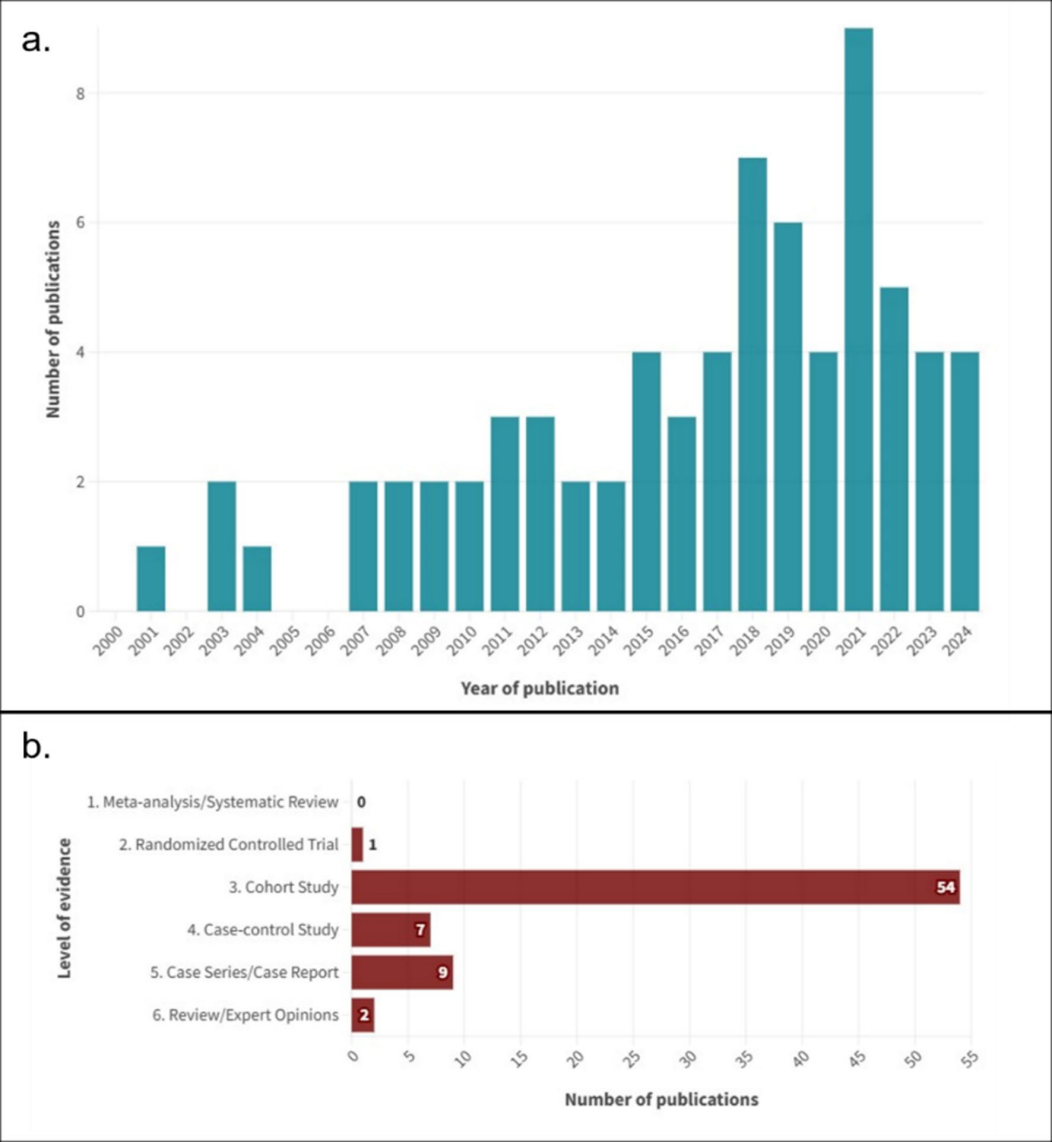
Characteristics of the Included Studies. (**A**) Number of studies published yearly from 2000 to 2024. (**B**) Level of evidence according to the evidence pyramid for medical studies

**Fig. 3:**
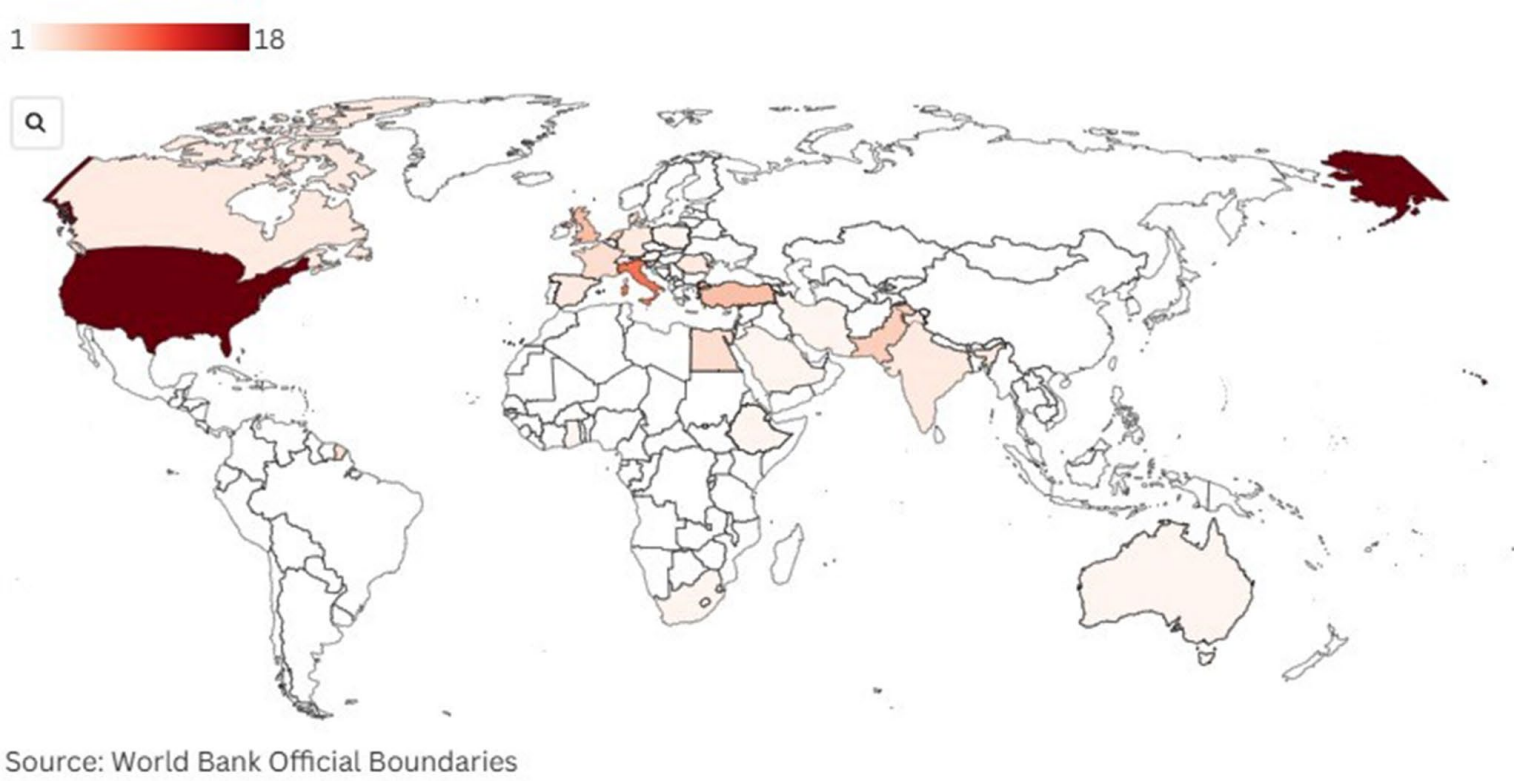
World map highlighting countries with publications on emergency incisional hernia repair

The characteristics of the included studies are summarized in Figs. 2 and 3. The studies were published between 2001 and 2024, showing a progressive increase over time, with the most prominent peak in 2021. Most of them were cohort studies (*n* = 54, 73.97%), followed by case series/case reports (*n* = 9, 12.33%), case-control studies (*n* = 7, 9.59%), narrative reviews (*n* = 2, 2.74%), and one randomized trial (1.40%). The geographic distribution of the studies showed a significant concentration in high-income countries, particularly the United States, which stands out as the country with the highest count (*n* = 18, 24.66%). Countries in Western Europe showed an intermediate number of publications (*n* = 28, 38.36%), highlighting Italy (*n* = 9, 12.33%) and the United Kingdom (*n* = 5, 6.85%). In contrast, fewer studies emanated from low and middle-income countries, such as those from Africa (*n* = 5, 6.85%), Southeast Asia (*n* = 2, 2,74%) and Latin America (*n* = 0). The sample sizes in the included studies varied widely, ranging from 1 (case reports) to 43,819 participants, with a median of 203 (64, 588). Of the 177,489 patients described in the included studies, 59,641 were explicitly described as emergency incisional hernia patients (33.60%).

**Fig. 4:**
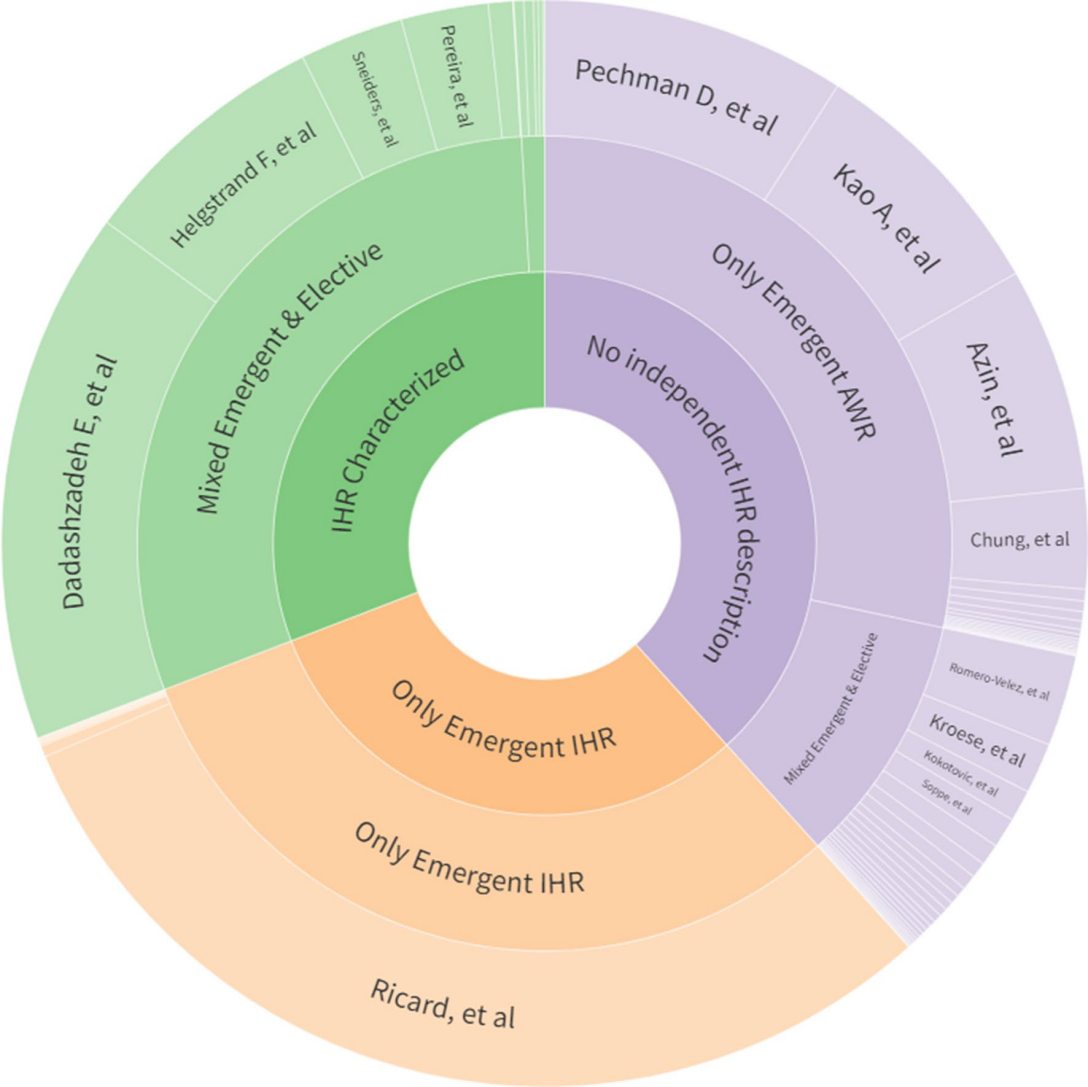
Types of approaches to emergency incisional hernia repair in literature, sized by the sample size of the included studies

**Fig. 5:**
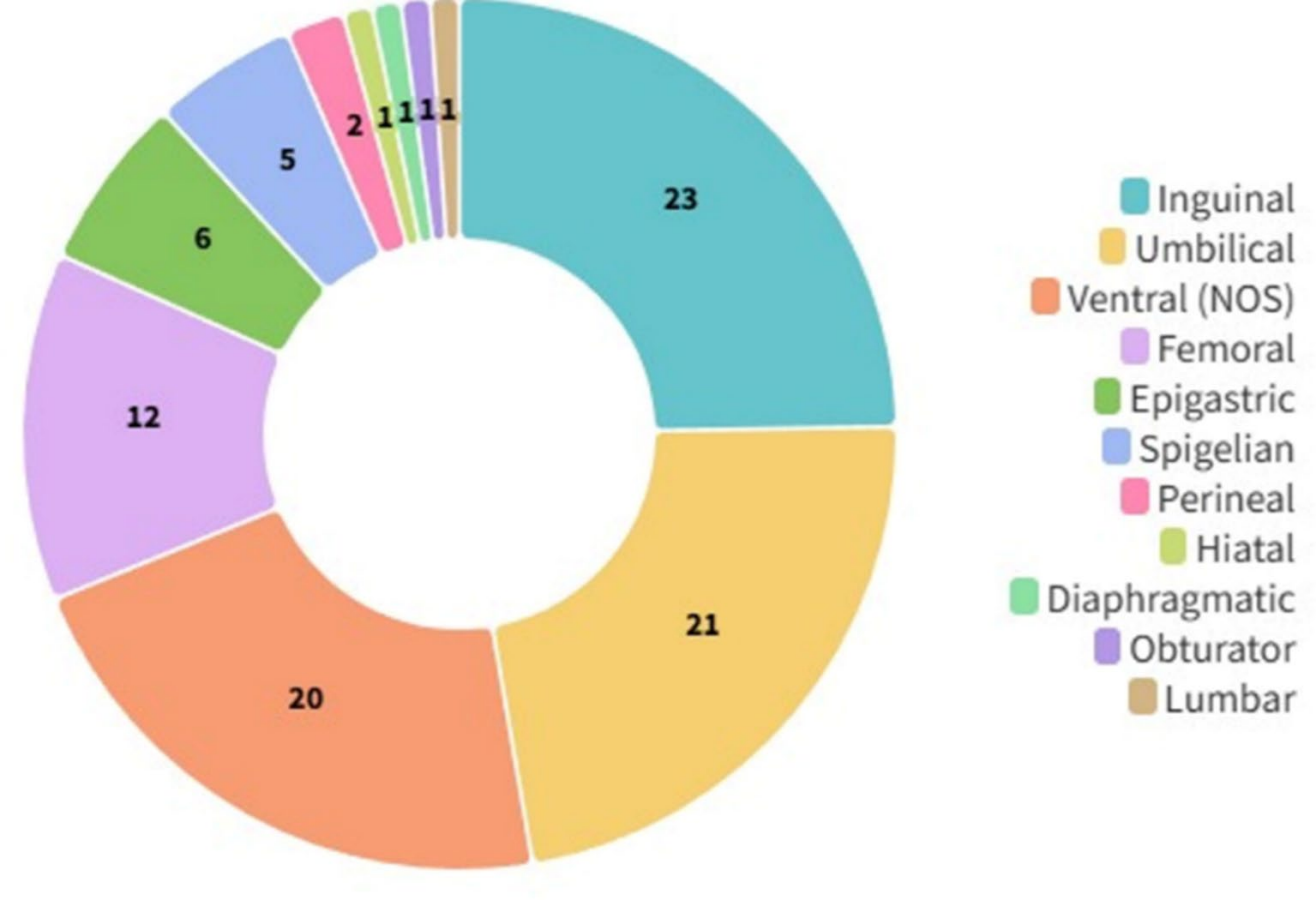
Types of hernias included in studies involving patients undergoing emergency incisional hernia repair. Values represent absolute frequencies

Three different types of approaches were identified in the literature (Fig. 4): (1) Studies specific to the context of emergency incisional hernia (*n* = 12, 16.43%); (2) Studies reporting emergency hernia surgery, including incisional hernia repair, and characterizing this group independently (*n* = 14, 19.18%); and (3) Studies reporting emergency hernia surgery, including incisional hernia repair, but not characterizing the IHR group (*n* = 47, 64.38%). Among the most common hernia types included in the non-context-specific studies, along with incisional hernias, were primary ventral hernias (*n* = 41, 56.16%), followed by inguinal/femoral hernias (*n* = 23, 31.51%) (Fig. 5).

### Primary focus areas

**Fig. 6:**
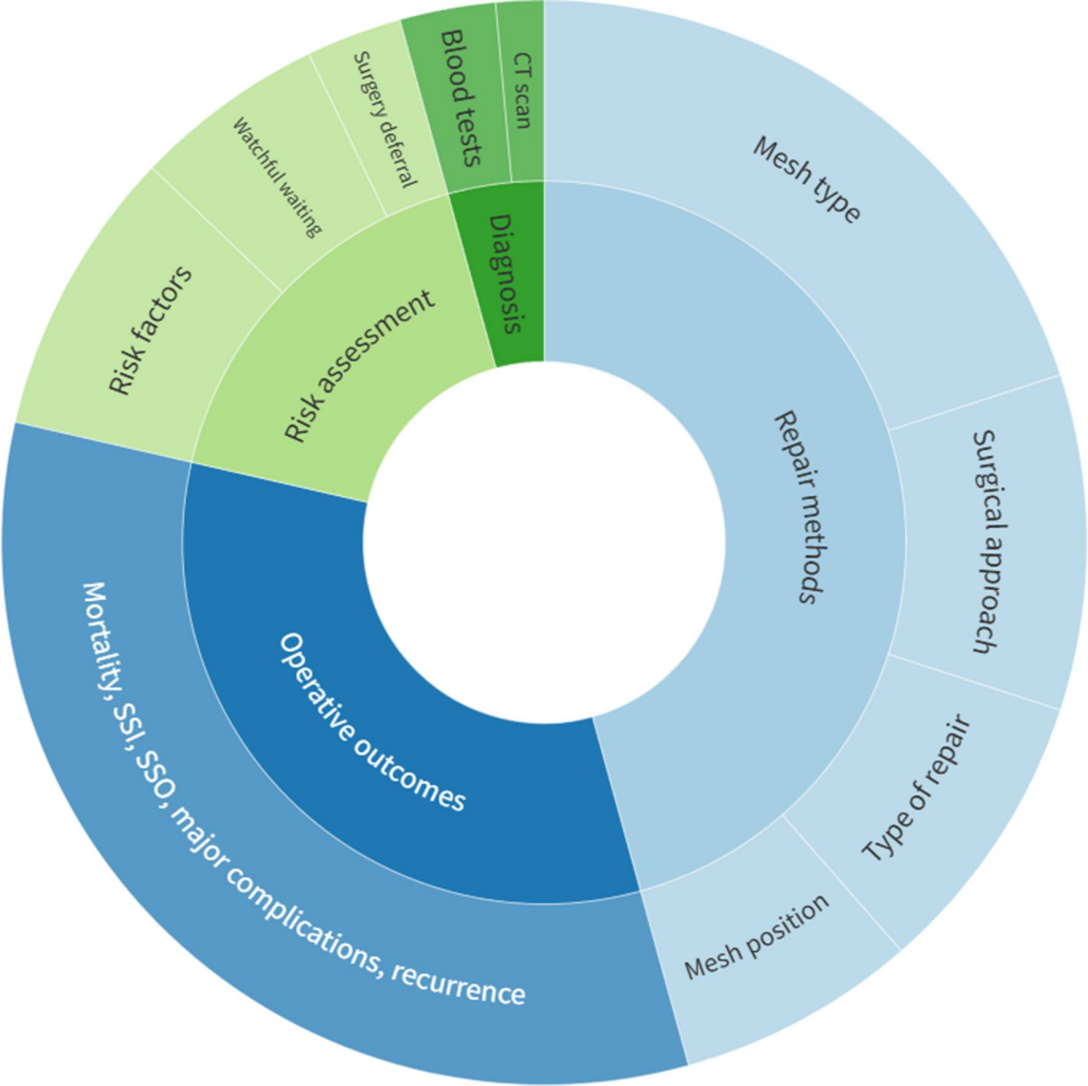
Subject areas of the included studies

**Fig. 7:**
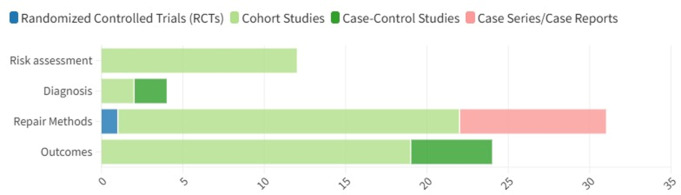
Study design per subject area

The studies were categorized into four primary focus areas: risk assessment, diagnosis, repair methods, and outcomes. Those covering multiple topics were categorized under all pertinent areas. The most frequent primary focus was on the repair methods (*n* = 35, 47.95%), followed by operative outcomes (*n* = 23, 31.51%), risk assessment (*n* = 12, 16.44%), and diagnosis (*n* = 4, 5.48%) (Fig. 6). The level of evidence for each subject is illustrated in Fig. 7.

### Risk assessment

Among the studies addressing the risk profile for emergency IH, three key topics emerged: the risk factors associated with emergency presentation, the risk for patients under watchful waiting management, and the risk associated with deferring surgery in requiring emergency hernia repair.

#### Risk factors

Most studies evaluating factors for emergency hernia repair were prospective and registry-based cohorts. The most common factors explored, besides sociodemographics, were comorbidities (individually and using the Charlson comorbidity index), hernia size and location, and computer tomography (CT) findings [13, 33, 64, 65, 74, 91]. Their analyses reflect that older age, female sex, African American race, active smoking, comorbidities (such as obesity, diabetes mellitus, heart failure, COPD, and a Charlson comorbidity index ≥ 2), and a higher ASA classification (III-IV) may increase the risk of requiring emergency IHR [13, 64, 74]. Additionally, specific clinical and CT findings have been identified that may increase the risk in both incisional and primary ventral hernias. These include the presence of small bowel within the hernia sac, a smaller angle between the hernia sac and the fascia (< 30–91º), and larger hernia sac sizes (sac height > 3.25 cm) [33, 91]. Regarding hernia size, studies have shown conflicting results. A large prospective study based on the French Hernia-Club registry found that wider incisional hernia defects (≥ 3 cm) are associated with a higher risk of incarceration [64]. Conversely, a recent Swiss study using the HerniaMed database reported significantly smaller mean incisional hernia sizes in patients undergoing emergency repair [65]. Additionally, another recent study found that a reduced fascial defect width was associated with a lower risk of incarceration [91].

#### Watchful waiting

Regarding watchful waiting (WW), few retrospective studies coming from Europe (*n* = 2) and the USA (*n* = 1) have addressed the effect of this treatment modality in IH patients, with rates varying largely among studies, from 41–78.1% [31, 38, 69]. Common indications included the absence of symptoms and comorbidities [38, 69]. Additional reasons were refusing surgery and large hernia sizes [69]. The cross-over rate was reported to be between 12.1% and 33%, with 1.7–7.7% of the patients requiring an emergency procedure during a maximum median follow-up of 68 months [38, 42, 69]. Complications from this approach after an emergency procedure included a significantly higher incidence of bowel perforation, postoperative fistula, and death compared to an elective procedure [31, 69]. Currently, an ongoing clinical trial in Germany (NCT01349400) evaluates WW vs. surgical repair in asymptomatic and oligosymptomatic (NAS < 3) incisional hernias in terms of pain and discomfort, patient satisfaction, quality of life, and frequency of incarceration during 24 months of follow-up.

#### Surgery deferral in the acute setting

Articles addressing surgery deferral in the acute setting (defined as emergency department discharge for acutely symptomatic hernias) are lacking specifically for IH. The current information on this matter comes from two American retrospective cohort studies describing outcomes after emergency department discharge, including ventral and inguinal hernias, with 2.7% and 10% of the patients having an incisional hernia, respectively [67, 71]. Reasons for this indication were related to medical comorbidities (38%), followed by patients’ refusal of surgery (26%) and obesity (10%). Furthermore, a common issue encountered during deferral of a surgical repair of abdominal hernias is the high rate of loss in follow-up, which is higher than 50% in the American cohorts, mainly accounting for the uninsured population. Complications associated with emergency management after surgery deferral included exploratory laparotomy and bowel resection [71].

### Diagnosis

Three retrospective studies evaluated the diagnostic strategies for complicated hernias, including a variable proportion of IH patients (3.9%, 14%, and 100%). Two studies examined the predictive value of preoperative blood tests for bowel resection, identifying that the neutrophil rate, neutrophil-to-lymphocyte ratio, and platelet-to-lymphocyte ratio were significantly higher in patients with this outcome [34, 39]. Moreover, one study evaluated the impact of preoperative CT scans on surgical delay and complications, identifying a significant association between the use of this imaging modality and delayed surgery, bowel resection, and ICU admission [35].

### Repair methods

Within this category, the following subcategories were identified: surgical approach, type of repair, mesh type, and plane for mesh placement.

#### Surgical approach

##### Laparoscopic vs. open repair

Of the five studies comparing open vs. laparoscopic surgery in the emergency setting, four were based on the ACS-NSQIP database with overlapping years (from 2006 to 2019), describing ventral hernia repair and including IH patients in unclear proportions [26, 32, 75, 92]. The utilization of laparoscopy in all incarcerated and strangulated ventral hernia repairs increased over time [92]. Patients undergoing an open approach were found to be older, more comorbid, and septic at the time of surgery than those treated through a laparoscopic procedure [26, 32]. A one-to-one coarsened exact matching analysis revealed that, while the laparoscopic approach was significantly associated with shorter hospital stays and a lower rate of 30-day wound morbidity, missed enterotomies were more common [75]. In the only study that exclusively compared the two approaches in IH patients, a retrospective cohort analysis was conducted to evaluate short-term outcomes between the laparoscopic Intraperitoneal Onlay Mesh (IPOM) technique and open retromuscular mesh repair. While describing larger defects in the open repair group, they identified that wound infection rates, analgesic requirements, and hospital stay were lower in the laparoscopy group, with no differences in operative time and overall complications [77]. A case series with 21 emergency IH patients managed with the IPOM technique also supported the use of this approach [28]. Finally, a case report highlighted the potential feasibility of the e-TEP technique in this context [52].

#### Robotic surgery

One retrospective study has described the robotic approach (IPOM, Transabdominal Preperitoneal (TAPP), and Totally Extraperitoneal (TEP) techniques) for emergency ventral hernias in a single-arm cohort of 34 patients (73.5% incisional hernias) [27]. Most of them were females, obese, and had an ASA 3 status, with a median APACHE-II of 6.5. Most hernias were midline umbilical and infraumbilical and were classified as non-contaminated (grade 2) according to the modified Ventral Hernia Working Group classification (mVHWG, 82.4%). A medium defect size (4–10 cm) represented 55.9% of the patients. The mean operative time was 139 min, with 20.5% and 11.7% of patients experiencing minor and major complications, respectively. One hernia recurrence was identified during a mean follow-up of 20.5 months.

#### Type of repair

Three publications compared suture vs. mesh repair in the emergency setting; of these, only one was exclusive to IH. In this retrospective Italian study from 2009, which did not describe the demographic and clinical characteristics of the two groups, bowel resection was more frequent in the suture repair group (26.79% vs. 18.18%). Additionally, the suture repair group had higher rates of surgical site infection (46% vs. 14%) and hernia recurrence (16% vs. 3%) during a 6-month follow-up [41]. In the remaining two studies, incisional hernia represented less than 20% of the included population [53, 83]. Patients treated with tissue repair were more likely to have peritonitis, ascites, and an ASA 4 at presentation. Similarly, strangulation of the bowel was more common in the tissue repair group, leading to a significantly higher rate of bowel resection and use of drains. Patients in the mesh repair group had a significantly lower incidence of SSI, postoperative sepsis, and ICU/hospital stay than those in the suture repair group. After multivariate analysis, bowel resection [53], longer operative times (> 2 h), and CDC class-IV wounds, but not the type of repair, were significantly associated with a higher risk of surgical site infection [83]. On the other hand, sepsis was the only factor affecting mortality.

One prospective cohort study evaluated the outcomes of mesh repair in emergency hernias with vs. without bowel resection [89]. Of the total sample size, 17.79% of the patients had an incisional hernia, and for these cases, an onlay polypropylene mesh repair with a prophylactic drain was utilized. Patients requiring a bowel resection had significantly longer hospital stays; however, no difference was found in wound infection rate, postoperative morbidity, mortality, and hernia recurrence between the two groups [89]. Conversely, a retrospective cohort, including only patients treated with permanent synthetic mesh and considering only emergency IH patients, described high wound complication rates (31%), which were even higher in patients requiring bowel resection (38.46%) [30].

#### Mesh type

Most studies addressing the hernia repair methods described the use of synthetic mesh, most of which were retrospective studies [24, 30, 41, 42, 45, 52, 59, 73, 74, 77, 84]. However, studies comparing different mesh types, in emergency IH, were scarce: A randomized trial compared standard polypropylene (pp) mesh with pp-based composite mesh in patients with complicated large ventral hernias (> 10 cm, 56.7% incisional). The study reported longer operative and mesh fixation times with the standard mesh but found no differences in wound complication rates between the two groups [37]. An additional single-arm retrospective study also described low wound infection rates (5.71%) using pp mesh in contaminated large incisional hernias [66].

A retrospective multicenter cohort study described the use of poly-4-hydroxybutyrate (P4HB, biosynthetic) mesh in contaminated IH [6]. 41% of the patients were treated in an emergency, and 79% were classified as grade 3 (contaminated) according to the modified Ventral Hernia Working Group (mVHWG) classification. One or more postoperative complications were seen in 46.5% of the patients, with a mesh explantation and mortality rate of 4.2% each. Additionally, a 12,4% recurrence rate was reported after one year of follow-up. A multivariate analysis showed that the emergency setting was an independent risk factor for major complications.

Two studies evaluated the use of biological meshes [51, 90]. Both were case series studies, one including three (swine dermal non-crosslinked collagen prosthesis) [51], and the other seven (porcine dermal collagen graft) patients with emergency IH [90]. Wound complications were identified in two cases in the first study, and no recurrences or mortality were described during a mean follow-up of 11.1 months.

#### Plane for mesh placement

Most of the included studies described the use of onlay mesh positioning in the emergency scenario (*n* = 13), followed by the retromuscular location (*n* = 11). However, only a few studies aimed to compare outcomes according to the plane for mesh location within emergency incisional hernia patients. To remark, Juul et al. described in a large cohort an increased risk of reoperation with the retromuscular mesh positioning in this setting, in comparison with onlay placement, after multivariate analysis [13]. Other studies, including but not exclusive to this context and with smaller sample sizes, have not reported this association [6, 81]. The onlay mesh repair has also been found to be an acceptable approach for treating large and giant incisional hernias in this context [37, 47].

### Operative outcomes

Factors associated with adverse outcomes were the second most common focus in the included studies. These factors were primarily analyzed in relation to general morbidity and mortality, surgical site infections, prolonged hospital stay, readmission, and recurrence. Additional outcomes evaluated were bowel resection, surgical site occurrences, reoperation, ICU admission, postoperative pain, and quality of life. Overall, observational studies indicate that emergency incisional hernia repair is associated with higher complication rates compared to elective procedures [93]. An ongoing clinical trial (NCT05620121) is currently assessing the surgical outcomes of patients specifically undergoing emergency repairs for incisional hernias.

#### Morbidity (NOS) and mortality

Most studies addressing these outcomes included any emergency abdominal wall surgery, exhibiting variable proportions of patients with incisional hernias (15–100%). The mortality rate also varied among studies, from 2.3 to 33% in developing countries [54, 78]. A multicenter retrospective survey directly evaluated the emergency IH subgroup and found that age ≥ 80, male sex, higher white blood cell counts, creatinine levels, increased mean arterial pressure, and bowel resection were associated with increased mortality [34]. Among the studies non-exclusive to IH, increased age (≥ 70 years) [43], a longer duration of symptoms, ASA 3 and 4, and bowel resection were independent predictors of higher morbidity and mortality [46, 57, 68].

#### Surgical site infection (SSI) and prolonged hospital stay

A multicenter prospective cohort sub-study from the Management of Acutely Symptomatic Hernias (MASH) study, including a low proportion of incisional hernias (7.9%), identified an association between increased body mass index (BMI) and risk of SSI [29]. Additionally, two retrospective studies based on the ACS-NSQIP database, including but not exclusive to either emergency or incisional hernia repair, described BMI ≥ 30, ASA ≥ 3, insulin-dependent Diabetes mellitus, open surgical approach, and prolonged operative times as potentially associated with wound infection and prolonged hospital stay [36], while laparoscopic approach being a potential protective factor [62]. Additional potential factors associated with prolonged hospital stay included female sex and duration of surgery [63].

#### Readmission and recurrence

A retrospective analysis of the Nationwide Readmission Database in the USA, focusing exclusively on patients who underwent emergency IH repair from 2016 to 2018, identified through multivariate analysis that mesh repair, particularly biological mesh repair, and large bowel resection were independent predictors of 30-day readmission [60].

In a separate retrospective cohort study, postoperative complications were found to be independent predictors of hernia recurrence in patients treated with laparoscopic ventral/incisional hernia repair, including those managed in an emergency setting [49].

### Focus on specific populations

#### Cirrhotic patients

Two studies (one retrospective cohort study and one case series) specifically addressed the cirrhotic population, including patients with emergency IHR [24, 76]. The cohort study aimed to compare elective vs. emergency incisional hernia repair using an open-inlay polyester mesh repair. It identified significantly longer hospital and ICU stays, increased seroma formation, and a higher recurrence rate in patients treated in the emergency setting, with a mortality rate of 40% [24]. In the case series, mixed abdominal wall hernias were included, with 16.69% being incisional hernias and 55.40% of the patients treated in the emergency setting. The surgical approaches varied, including both suture and mesh repairs, as well as open and laparoscopic techniques. The mortality rate differed significantly according to the Child classification system, ranging from 4.1% in Child A cases to 72.72% in Child C cases [76].

No other study was dedicated to a specific comorbidity or relevant clinical condition. However, in the review of patients’ characteristics, four studies reported more than 90% of their included patients with a BMI in the range of obesity [26, 36, 37, 73], and one described their experience with a patient with multiple hernia defects [52].

### Pooled data description

Based on the general data provided by each article, several aspects of data presentation and description were identified, showing a high heterogeneity in general.

Among the studies, the most frequently reported medical history information included Type 2 Diabetes mellitus diagnosis (*n* = 15), previous hernia repair (*n* = 12), COPD (*n* = 11), smoking (*n* = 10), obesity (*n* = 9), cirrhosis (*n* = 8), cancer (*n* = 5), immunocompromise (*n* = 3), bleeding disorders (*n* = 2), multiple hernia defects (*n* = 4). Considering the hernia defect, the most common trend was the lack of reporting of the hernia location (either using the EHS classification or the abdominal areas) (*n* = 64, 94.11%). Information regarding hernia size was lacking in most of the studies (*n* = 46, 67.64%), and in the remaining, it was widely heterogeneous, with articles describing it with the EHS classification, others as means and standard deviations, and others reporting medians and interquartile ranges. Additionally, a clear description of hernia measure methods was only available in four studies. Among the 22 studies that described the hernia diameters (*n* = 32.35%), six studies (27.27%) included patients with small defects (< 4 cm), nine (40.91%) patients with medium-size defects (4–10 cm), and seven (31.82%) patients with large and giant defects (> 10 cm) according to the EHS classification.

### Characteristics of studies describing the emergency incisional hernia repair population

From the 24 studies that provided exclusive information on emergency incisional hernia repair, the total pooled number of patients included was 46,248 (median = 63, Q1 = 28, Q3 = 202). Of note, the study of *Ricard et al.* represented an outlier, accounting for 94.75% of the total sample size [60]. This study evaluated readmission trends and associated factors in emergency incisional hernia patients using the U.S. National Readmissions Database, identifying that mesh use was associated with higher odds of 30-day readmission (synthetic (OR 1.07, 95% CI 1.00–1.14), biologic (OR 1.26, 95% CI 1.06–1.49)) compared to suture repair. Additionally, patients with biologic meshes were found to have the highest readmission rates. However, as for its nature, the present study lacked detailed clinical characteristics and preoperative data.

#### General patient characteristics

The studies’ reports of the patients’ demographic and clinical characteristics varied widely and were highly heterogeneous. As much as the data registered allowed, a pooled analysis of these characteristics was conducted.

The included patients were most frequently women (Pooled Proportions [PP]: 63% [95% CI: 52-72%], 24 studies, I^2^:90%), elderly (Pooled Mean [PM]: 62.3 years [95% CI: 59–65 years], 15 studies, I^2^:89%), overweight/obese (PM of BMI: 33.4 kg/m^2^ [95% CI: 28–38 kg/m^2^], six studies, I^2^:96%), and with a high ASA class (PP ASA 3–4: 61% [95% CI: 48–74%], seven studies, I^2^: 78%). The pooled proportion of patients with Type 2 Diabetes mellitus among the studies was 27% (95% CI: 19-38%, five studies, I^2^: 72%), and active smoking was 24% (95% CI: 8-53%, six studies, I^2^: 97%). In addition, the pooled proportion of previous hernia repair was 29% (95% CI: 18-42%, eight studies, I^2^: 97%).

#### General treatment characteristics

Fourteen studies reported using mesh, with a high degree of heterogeneity regarding the proportion of patients that received a prosthetic repair. Among these, the lowest proportion of mesh use was reported in *Ricard C et al.* (68%), with seven studies reporting only emergency incisional hernia repairs with mesh. Moreover, seven studies described the mesh type used; three evaluated only the use of permanent synthetic mesh (*n* = 148 patients). At the same time, three included patients treated with synthetic or biological meshes (*n* = 29,796), and one described only using biological mesh (*n* = 4).

#### General postoperative outcomes

Among studies that reported patient longitudinal data, the median follow-up was three months (range: 1–12 months), with the most frequent adverse outcomes observed being: readmission (PP: 18% [95% CI: 12-26%], five studies, I^2^: 86%), SSI (PP: 12% [95% CI: 7-18%], six studies, I^2^: 35%), reoperation (PP: 8% [95% CI: 3-21%], five studies, I^2^: 92%) and mortality (PP: 4% [95% CI: 2-8%], 12 studies, I^2^: 95%), with a pooled proportion for any postoperative complication of 31% (95% CI: 18-48%, five studies, I^2^: 86%). No data regarding patients’ reported outcomes was identified.

## Discussion

The current literature on incisional hernias treated under emergency conditions clearly lacks robust evidence and standardization. The presence of a single RCT reflects the difficulty of conducting such research in a field where standardization is very difficult and patients arrive for surgical evaluation in various conditions, making controlling for confounders almost impossible, out of large-scale studies.

The pooled descriptive data reveal that emergency incisional hernias are most commonly observed in female, elderly, and comorbid patients. In terms of treatment, the open approach with synthetic mesh is the most employed technique. Notably, the overall postoperative complication rate for emergency incisional hernias was high, reflecting the challenging nature of managing these cases. Importantly, the definition of emergency hernia is not consistently provided in current literature. The different definitions of acutely symptomatic incisional hernia have created a heterogeneous group of publications in which it is also difficult to differentiate patients with incarceration, obstruction, or perforation on one side from those with adhesive small bowel syndrome associated with an incisional hernia on the other. The non-elective presentation of an abdominal wall defect has different implications on patients’ treatment and prognosis: a pathology mainly referred to a single anatomic area suddenly becomes a problem with systemic effects, requires fluid resuscitation and stabilization, and may or may not involve sepsis. The priorities of the treatment are directed at saving the patient’s life, preserving the bowel involved in the process, and then minimizing the need for further interventions by repairing the abdominal wall.

Methodologically, the first issue is represented by the fact that papers with a robust structure in the emergency IH setting are lacking (74% are cohort studies), as well as direct comparisons (9%) or unbiased evaluation. Accordingly, the analysis of this literature and the development of a treatment algorithm through PICO questions can be challenging, particularly for guideline developers. Nevertheless, it should be acknowledged that, in this area, the production of surgical clinical trials is two-fold complex. The first reason is connected to the well-known methodological problems typical of the surgical environment, which conflict with the conduction and results interpretation of trials [95]. The second is typical of the emergency area and is connected to time restraints that can interfere with the allocation of definitive treatment and endanger the life of the patient. Despite several methodologies that have been proposed, reliable studies are lacking or flawed by many biases.

The second methodological problem that we have encountered is connected to the heterogeneity of studies in terms of the type of defect enrolled. Almost 75% of studies dealt with a mixed group of patients operated on for an abdominal wall defect, irrespective of its nature, either primary inguinal, ventral, or incisional. This represents a major issue since inguinal hernias are treated according to defined procedures that have standard results, and the characteristics of the myopectineal orifice have principles of treatment totally different from those of other defects (surrounding bony structure and favorable anatomical planes). Moreover, pooling together primary ventral and incisional hernias frequently carries a major bias in interpreting results. It has been clearly shown in elective settings that IHs have unfavorable features and generally are more complex to repair: patients are older and more comorbid, defects have wider dimensions, are multiple (Swiss-cheese), adhesiolysis is frequently necessary, and the results of the repair are worse (longer LOS, more frequent complication, higher recurrence rates) [96]. The effect of the higher complexity is clearly amplified in emergency settings where mortality and complications rise, and the pooling with primary hernias introduces cases with similar presentations but more stable features. Accordingly, the presence of inguinal and primary ventral hernias can only mitigate the results and lead to an underestimation of the real burden of IH emergency treatment.

A third methodological problem arises from the type of outcomes, method of assessment and follow-up period chosen. Concerning outcomes, only preoperative surgical ones are analyzed, without any evaluation of patient-centered outcomes (PROMS) as already highlighted in the elective settings [97]. The method of outcome assessment is usually inconsistent and subjective, while the follow-up period is mainly focused on 3 months postoperatively.

In the present analysis, we have shown that the main concern for surgeons dealing with emergency IH repair is focused on technical aspects and factors predicting adverse outcomes. As shown in our analysis, the baseline risks of performing incisional hernia repair in the context of an emergency are 4% mortality and 31% morbidity, without possible distinction between aggravating patient, technical, and expertise factors. The armamentarium available for the surgeon is very limited and ranges from “damage control” strategies (open abdomen, planned incisional hernia) to suture repair or formal abdominal wall reconstruction (AWR).

The first factor that has a relevant impact on the choice of treatment is the patient’s condition in terms of stability and possible functional reserve. Information on patients’ stability was not possible to be retrieved. Nevertheless, our results suggest an elevated mean age and BMI in the emergency series; more importantly, patients presenting in the acute setting have a high ASA class on admission, explaining the possible increased risk of adverse events registered. The choice of treatment in this fragile cohort should be tailored, taking into consideration all the pre-existing conditions (diseases and medication) that cannot be preoptimized and will affect outcomes, reserving more aggressive strategies for the healthier and more stable subjects. No indication of a possible threshold to opt for treatment can be retrieved in the selected papers.

The second relevant variable in the management of acute IH is the presence and degree of bowel obstruction and the associated adhesive syndrome. It is known that extensive adhesive syndrome can be a factor of intra and postoperative complications as well as increased operative time. *Ten Broek et al.* showed that in the elective setting, adhesiolysis time longer than 30 min is a factor predictive of increased postoperative complications, higher rate of sepsis, and enterotomies [98]. Enterotomies and obstruction can change the strategy of repair by affecting the choice of material and/or the execution of a formal abdominal wall repair when the risk of a compartment syndrome is actual. This condition can only be worsened in emergency IHR. Of note is the fact that usually, incisional hernias are connected more frequently with adhesive small bowel in comparison to primary ventral, posing this type of problem more frequently.

The third relevant factor related to emergency IHR is the approach to a complex abdomen becoming acutely complicated. The complex abdomen has been defined by various experts. One of the first definitions was published by *Slater et al.* in 2014: in their description, the emergency setting was considered as one of the criteria connected with moderate complexity for the frequent association with hernia-complicating factors such as the presence of obstruction, previous meshes, infection, etc [99]. Recently EHS endorsed a revised definition of complex abdomen in which emergency setting disappeared and patient, hernia, abdominal wall and operation site-related factors were chosen with Delphi methodology by a group of experts as primary determinant of complexity [100]. Facing a complex abdomen in the emergency setting is a frequent situation that requires attention and expertise to be treated with success. This is particularly true when large defects and contamination are the dominant scenario of the emergency hernia. In this situation, the decision of whether to undertake the repair or adopt a conservative approach, the type of mesh, and the position can be challenging and expose the patient to a higher risk of septic complication and abdominal compartment syndrome. Interestingly, in the selected literature, data on hernia characteristics were reported in the minority of papers, a formal classification of defects is rarely retrieved, and a complete description of complexity is lacking, ultimately making it very difficult to evaluate criteria for choice of treatment.

Concerning details of treatment, most papers with a primary focus on treatment have, as their main aim, the analysis of different mesh types and minimally invasive approaches (robotic and laparoscopic) showing a sort of spin in emergency literature directed at the analysis of new therapeutical opportunities and their possible role without considering the actual clinical questions.

The use of prostheses in the context of emergency is a significant topic of debate. Factors such as the risk of colonization of synthetic prostheses, the degree of wound contamination, and uncertainties about the early behavior of absorbable meshes in contaminated fields complicate the evaluation of their effectiveness. Additionally, concerns about the risk of recurrence with biological meshes and ambiguities related to bio-like meshes prevent drawing definitive conclusions about their roles. In the current selection of articles, direct comparisons between different mesh types and their indications for use in emergencies were not available.

Minimally invasive surgery, even if feasible and possibly beneficial on general morbidity, by simply allowing inspection of the abdominal cavity, suffers from the fact that it can be accomplished in a highly selected group of stable patients where the obstruction is not already fully developed, and generalized peritonitis is not present. Moreover, the presence of intense adhesive small bowel syndrome can make this a challenging approach limited to experts. Accordingly, even if clinically relevant, the use of minimally invasive surgery addresses only a small proportion of the possible scenarios involved in an emergency and is currently recommended for early stages where a single band of adhesion is suspected [98].

The last update of inguinal hernia treatment guidelines from EHS has attempted to define on what is an emergency inguino-femoral hernia, pointing to the concept of the sudden change of the clinical status of the hernia (acute irreducibility – strangulation), abandoning the concept of chronic incarceration. Acute incisional hernia represents a more nuanced condition where time has relevance in both the development of obstruction and contamination and, finally, in treatment. If accepted as a generalization, the acute presentation of IH can be approximated to small bowel obstruction (SBO) caused by the IH or complicated by the IH in which surgery must take place in a timely fashion and where the repair of the abdominal wall gap is a complicating factor. Currently, a wide and inconsistent definition of emergency incisional hernia has been provided in the selected papers, explaining the extreme heterogeneity of cases collected and making it hard to draw an effective treatment algorithm. According to this concept, it is of interest that only two studies have assessed the role of CT specifically in the scenario of complicated incisional hernia, evaluating its possible impact on reducing treatment delay. Nevertheless, CT is considered the test of choice for evaluating patients with SBO [98]. This imaging technique improves the identification of high-grade obstructions and those that are unlikely to resolve non-operatively. Furthermore, CT scans have approximately 90% accuracy in predicting strangulation and the need for urgent surgical operations. This exam could represent the initial step in defining and stratifying patients with SBO and IH.

The first advantage of having a standardized classification could be the possibility of conducting homogenous studies with more reliable results that could allow data collection to inform guideline development, in which different scenarios are addressed with sufficient clarity to be reproducible by the general surgeon in the emergency setting. Other advantages include the possibility of exploring different treatments and their role being limited to certain subgroups only. Of interest could be the evaluation of non-operative management in stable patients with large defects, damage control strategies in unstable patients with SBO, and the role of suture and mesh repair in this context.

This scoping review has several limitations. Restricting the search to English-language publications may have introduced bias, potentially excluding relevant studies in other languages. The exclusion of conference abstracts and studies where full texts were unavailable could have led to the omission of potentially valuable data, particularly in a field with limited literature. Additionally, the focus on studies published between 2000 and 2024 ensured contemporary relevance but may have excluded earlier foundational research. While these limitations may affect the comprehensiveness of the findings, the review provides a detailed mapping of the existing literature on emergency incisional hernia repair and highlights key gaps for future research.

## Conclusion

This scoping review reveals a significant gap in evidence guiding the diagnosis and management of emergency incisional hernias. Key issues identified include inconsistent definitions of emergency presentation, limited characterization of hernia defects and clinical scenarios, and a lack of standardized outcome reporting. Current literature emphasizes that management in this setting should prioritize addressing immediate life-threatening conditions, such as bowel obstruction, perforation and sepsis, while minimizing unnecessary dissection to prevent the spread of infection or disruption of anatomical planes. Although comprehensive guidelines are not yet available, these principles underscore the need to balance effective intervention with harm reduction and highlight the critical need for further research to develop more standardized management strategies. To advance the field, future research should focus on developing a unified classification system for emergency incisional hernias, careful selection of reference standards, and better characterization of defect types and bowel involvement. Evaluating the role of imaging in decision-making could enhance preoperative planning and optimize treatment strategies. Further studies should also aim to analyse the complex interactions among patient conditions, hernia characteristics, and the degree of bowel involvement. This approach would help better define optimal treatment strategies for this frail patient population, which is at higher risk of complications. Given the difficulty of performing randomized trials in this context, multicenter-matched cohort studies and registry-based studies should continue to be promoted to support evidence-based guidelines for this challenging patient population.

## Electronic supplementary material

Below is the link to the electronic supplementary material.

Supplementary Material 1

Supplementary Material 2

Supplementary Material 3

Supplementary Material 4

Supplementary Material 5

Supplementary Material 6

Supplementary Material 7

Supplementary Material 8

Supplementary Material 9

Supplementary Material 10

Supplementary Material 11

Supplementary Material 12

Supplementary Material 13

Supplementary Material 14

Supplementary Material 15

Supplementary Material 16

Supplementary Material 17

Supplementary Material 18

Supplementary Material 19

Supplementary Material 20

Supplementary Material 21

Supplementary Material 22

Supplementary Material 23
