# Supplementary material for: Mapping the therapeutic landscape in emergency incisional hernia: a scoping review
Source: Hernia. 2025 Feb 18;29(1):102. doi: 10.1007/s10029-025-03278-y (PMC11836210; doi:10.1007/s10029-025-03278-y)
Supplement: Supplementary file 1 — Supplementary Material 1 [file 10029_2025_3278_MOESM1_ESM.docx]

**Supplementary material**

**Mapping the Diagnostic and Therapeutic Landscape in Emergency Incisional Hernia: A Scoping Review**

*Brief Title:* Mapping Emergency Incisional Hernias: A Scoping Review

**Journal: Hernia**

1. **Search Strategies**
   1. **Search Strategy in PubMed MEDLINE**

("emergen*" OR "urgen*" OR "Emergencies"[MeSH]) AND ("treatment" OR "treat*" OR "repair*" OR "manage*" OR "surger*" OR "surgical" OR "Surgical Procedures, Operative"[MeSH] OR "Herniorrhaphy"[MeSH]) AND ("complicat*" OR "incarcerat*" OR "strangulat*" OR "Hernia, Ventral/complications"[MeSH]) AND ("hernia" AND "incisional") AND ("2000/01/01"[Date - Publication] : "3000"[Date - Publication])

- 1. **Search Strategy in SCOPUS**

TITLE-ABS-KEY ( emergen* OR urgen* OR "Emergencies" ) AND TITLE-ABS-KEY ( treatment OR treat* OR repair* OR manage* OR surger* OR surgical OR "Surgical Procedures, Operative" OR "Herniorrhaphy" ) AND TITLE-ABS-KEY ( complicat* OR incarcerat* OR strangulat* OR "Hernia, Ventral complications" ) AND TITLE-ABS-KEY ( hernia AND incisional ) AND PUBYEAR > 1999 AND PUBYEAR < 2025
